# Contact Geometry Affects Lesion Formation in Radio-Frequency Cardiac Catheter Ablation

**DOI:** 10.1371/journal.pone.0073242

**Published:** 2013-09-23

**Authors:** Neal Gallagher, Elise C. Fear, Israel A. Byrd, Edward J. Vigmond

**Affiliations:** 1 Department of Electrical and Computer Engineering, University of Calgary, Calgary, Alberta, Canada; 2 St. Jude Medical, Atrial Fibrillation Technology Development, St. Paul, Minnesota, United States of America; 3 LIRYC Electrophysiology and Heart Modeling Institute/Laboratoire Institut de Modélisation, Université Bordeaux 1, Pessac, France; University of Minnesota, United States of America

## Abstract

One factor which may be important for determining proper lesion creation during atrial ablation is catheter-endocardial contact. Little information is available that relates geometric contact, depth and angle, to ablation lesion formation. We present an electrothermal computer model of ablation that calculated lesion volume and temperature development over time. The Pennes bioheat equation was coupled to a quasistatic electrical problem to investigate the effect of catheter penetration depth, as well as incident catheter angle as may occur in practice. Biological experiments were performed to verify the modelling of electrical phenomena. Results show that for deeply penetrating tips, acute catheter angles reduced the rate of temperature buildup, allowing larger lesions to form before temperatures elevated excessively. It was also found that greater penetration did not lead to greater transmurality of lesions. We conclude that catheter contact angle plays a significant role in lesion formation, and the time course must be considered. This is clinically relevant because proper identification and prediction of geometric contact variables could improve ablation efficacy.

## Introduction

Radio-Frequency (RF) ablation is a minimally invasive interventional technique that has demonstrated efficacy in treating cardiac arrhythmia, specifically atrial fibrillation (AF) [1]. In the procedure, a catheter is advanced to the target site in the heart through a venous access point. RF current heats the myocardial tissue, via the Joule effect, to temperatures in excess of 50°C at which point cellular necrosis occurs, resulting in a permanent loss of electrical excitability [2]. In the case of atrial ablations, the goal is often to isolate the pulmonary veins where the sources of AF reside.

The success of this procedure is entirely dependent on full transmural lesion formation [3] for electrical isolation. If too much energy is applied, there can be serious complications like perforation of the atrium, tamponade or embolisms [4]. Of all the factors that influence lesion formation, local catheter-endocardial contact geometry (penetration depth and incident angle) is the least well controlled due to a lack of soft tissue contrast in the fluoroscopy images used to track catheter location.

There is a large amount of data illustrating the important effect that catheter/endocardial contact has on electrical coupling[5], naturally occurring cooling, heat accumulation, maximum temperature, the transient response of each, and the resulting lesion formation [1], [3], [5]–[14]. In addition, preliminary studies have shown that there are significant differences in initial measured electrical impedance depending on catheter/tissue angle even at the same penetration depth [15]. Despite this, and the fact that unknown endocardial contact geometry is a well known limitation of the procedure [16], how lesion formation is affected by the incidence angle of the catheter requires further elucidation.

The purpose of this study is to investigate the impact that penetration depth and catheter angle have on what are widely recognized as key empirical factors for procedural success and safety (i.e. lesion depth, volume and maximum temperature). In order to accomplish this goal, an electrothermal computer model of atrial ablation was constructed and the resulting temperature distributions for various catheter angles and penetration depths were analyzed. This paper describes the implementation of the physical, electrical and thermal models, as well as how the data are generated. Finally, given computed temperature distributions, approximate lesion boundaries were fitted and trends analyzed.

## Methods

### Computer Simulations

#### Model Construction

**Figure 1:**
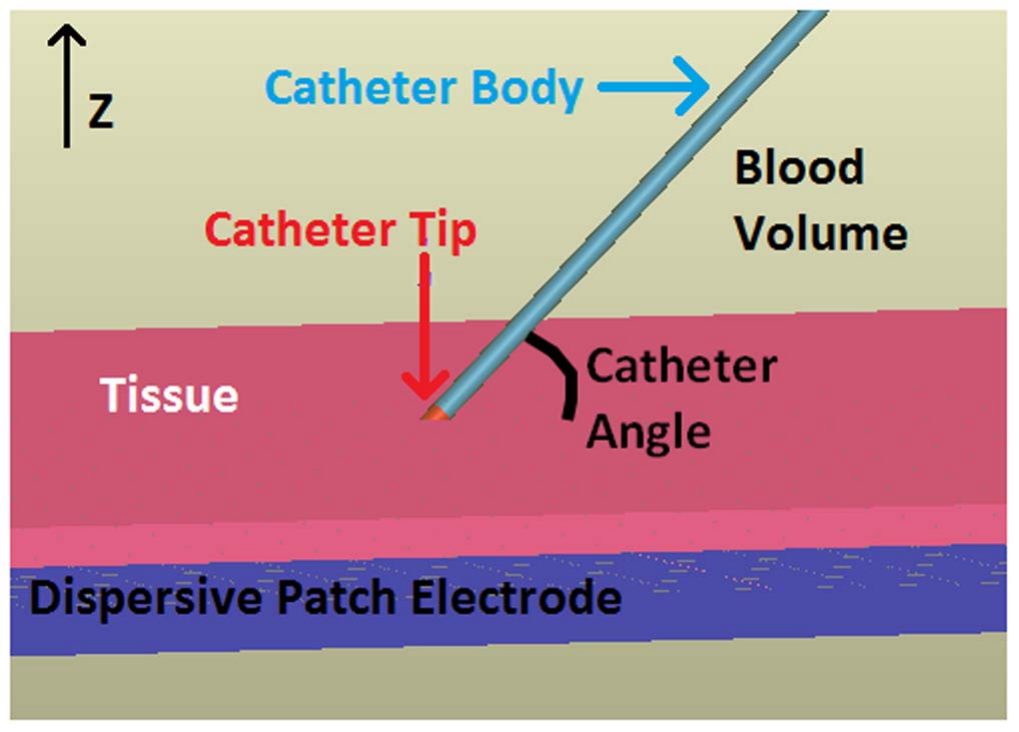
Sample ablation model setup. Dispersive patch electrode is on bottom in blue, catheter body is in light blue, catheter tip is red and myocardial tissue is pink. Blood fills the rest of the volume. Scenario shown has catheter at 3.2° angle (calculated from the tissue surface normal).

The computation of heat propagation throughout the tissue during an ablation requires the solution of an electro-thermal problem. Both the electrical and thermal portions of the models were constructed using SEMCAD (Schmid & Partner Engineering AG, Switzerland). A slab of tissue (100×100×4 mm thick) was suspended in a blood solution measuring 150×150×75 mm with the ablation catheter contacting the center of the slab. A dispersive patch electrode (the reference) was placed 4 mm below the tissue. The catheter to be modeled has a platinum-iridium and a polyurethane cylindrical extrusion (the body of the catheter), representing a 7 French, 4 mm 485 kHz ablation catheter. Since the nature of situating the catheter can lead to any number of contact scenarios, electrical and thermal simulations were run for catheter/tissue interface angles of 15°, 30°, 45°, 60°, 75° and 90° and penetration depths of -4 (in the blood pool), 0.04, 0.8, 1.2, 2.4, 3.2 and 4 mm (fully engaged in the tissue). Catheter angles are defined relative to the surface tangent meaning that an angle of 90° is perpendicular to the surface of the tissue (Figure 1).

#### Electrode-Tissue contact

The physical environment in which ablation occurs is quite complex and the exact surface layout differs from patient to patient. Irregularly shaped trabeculated muscular tissue on the endocardium makes modelling the physical anatomy challenging. We approximated this complex tissue structure with a homogeneous smooth layer of tissue. Since the complex geometry of the atria abounds in crisscrossing muscular ridges, it is possible that the catheter settles into a “valley” of these ridges and becomes fully engaged with the tissue. Thus, it was assumed that all of the penetrating portion of the catheter made contact with the tissue.

#### Electrical Problem

The electrical problem must be solved for the entire solution space in order to obtain the current density distribution. Given the low frequency of interest, the Electroquasistatic assumption could be used, allowing decoupling of the electric and magnetic fields by setting the time varying magnetic field to zero.

A Finite Element (FE) based low frequency solver was used for the electrical problem. Accordingly, the Laplacian (equation 1) was solved everywhere in the solution space:
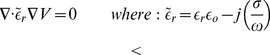
(1)


Zero flux (Neumann) boundary conditions were imposed at all edges and the blood region was chosen sufficiently large to prevent boundary effects. The electrical properties of the tissue and the blood, permittivity and conductivity, were calculated using multiple Cole-Cole dispersions[17], evaluated at 485 kHz (see table 1).

**Table 1:** Material Properties Used in Electrical and Thermal Problems.

Material	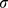	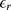	c	k	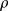
	S/m	-	J/kg K	W/m K	kg/m^3^
Blood	0.7459	4227.12	3600	0.492	1047
Myocardium	0.2790	3332.32	3200	0.5367	1063
Pt-Ir	4e6	NA	132	71	21.5e3
Polyurethane	4.7e-4	2.54	NA	NA	NA

#### Thermal Problem

With the current density distribution obtained, the same mesh was reused to solve the thermal problem. The accumulation and propagation of heat over time is important for analysis so a steady-state solution is insufficient. A conventional FDTD solver was employed to solve the standard parabolic Pennes bioheat equation for conduction:
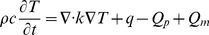
(2)


where 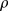 is density, 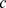 is specific heat capacity, 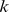 is thermal conductivity, and 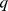 is the source term from the RF power deposition. Since the tissue is thin and generally, ablation sites are far from major blood vessels, blood perfusion heat loss (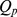) in the myocardial tissue and the metabolic heat generation rate (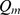) were neglected. The physical material properties used in the model are shown in Table 1 and were taken from literature [18] [19] [8] [20] [21] [7] [22] [13] [10].

**Figure 2:**
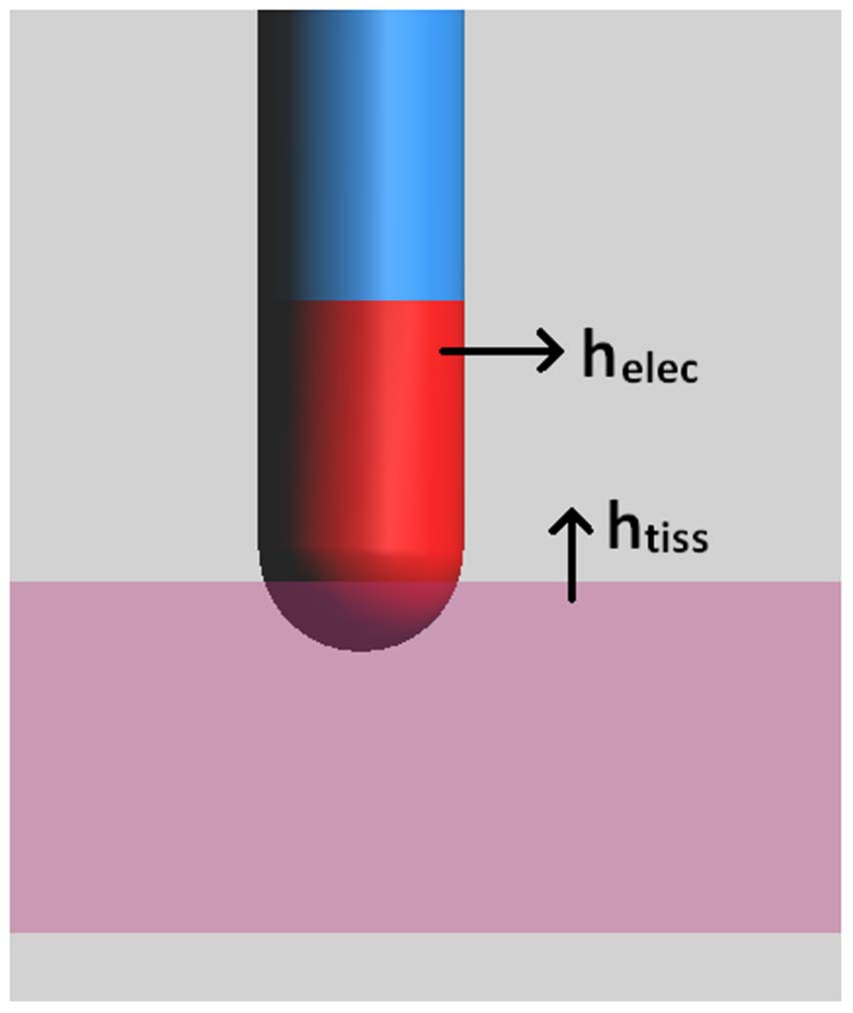
Sample ablation model setup. Sample ablation model illustrating the two convective interfaces that exist in the model.

The temperature of the entire model was initialized to 37°C. Increases in heat were due to the resistive heating of the tissue. The cooling aspect of the problem also had to be addressed. Particularly significant in endocardial ablation is the rate at which heat is lost to the blood that is continuously being exchanged inside the atrium. Convective film coefficients were implemented as boundary conditions at the interface between the catheter tip and the surrounding blood pool (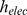) and the interface between the myocardial tissue and the blood pool (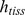) as illustrated in Figure 2.

Previous work used 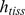 film coefficients calculated using average blood flow velocities measured in the heart using ultrasound, or from in-vitro studies featuring convection coefficients measured in rubber and plastic models, with values in the range of 44–2500 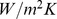 [13] [10] [11] [6]. It is not surprising that the range of values is so large and there are quite a number of valid physiological explanations including varied heart rate, varying atrial condition during the procedure and drastically different blood flow conditions at different ablation sites. The value for 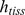 used in this study was 5350 W/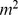
K and was taken from an in-vivo study using measurements of swine endocardial convective heat transfer coefficients taken on the lateral wall of the left atrium [23]. Using this experimentally measured value for 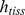 was logical given that the other lower values were computed using assumptions of laminar flow; the blood flow in the atria is known to be quite turbulent and therefore lead to a significantly higher convective coefficient.

The values used for 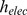, however, depended on a multitude of factors and had to be calculated. Other attempts to address this complex issue have thus far relied on laminar flow assumptions [11], [24], which is not applicable in the left atrium. To determine the convective coefficient of the electrode/blood pool interface, a Churchill-Bernstein correlation was used to calculate the Nusselt number. This correlation is typically used in scenarios which feature a cylindrical solid in cross-flow in which a large range of Reynolds and Prandtl numbers are possible, and where the flow is turbulent [25]. The Nusselt number (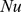) is calculated from
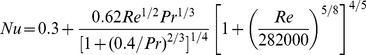
(3)


with the Reynolds number, 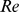, of the fluid flowing around the catheter computed as
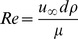
(4)


where 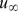 refers to the stream flow, 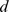 is the catheter diameter and 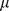 is the dynamic viscosity of blood. Next, the Prandtl number (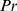) is calculated using only properties inherent to the blood itself:
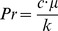
(5)


Finally, the convective film coefficient 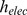 is computed:
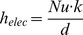
(6)


Using a stream flow velocity (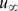) of 0.244 m/s, measured using Doppler ultrasound[11], the convective film coefficients are calculated and compiled in table 2.

**Table 2:** Convective Film Coefficients Used in Model.

Interface	Symbol	h
	W/*m^2^ K*	
Catheter tip/blood pool	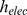	5482.3
Myocardium/blood pool	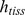	5350.0

#### RF Ablation Mode and Analysis Metrics

While there are quite a few ablation modes that are employed clinically, experimentally and in simulations, for this study a constant voltage mode was implemented. This decision was made for two main reasons:

- Natural phenomena are not as easily observed with compensatory schemes (such as constant temperature, constant power or irrigation). The goal of this study is to assess the effect that contact has on resulting lesion formation not to relate to actual procedural success.
- Ease of implementation.

Correspondingly, the peak voltage was set to 23V, roughly corresponding to a initial power of 5W (at the higher contact scenarios). The thermal simulation was run for 60 s (simulation time). Myocardial injury is known to occur at ≈50°C but there are no established data defining the time/temperature relationship above 50°C; therefore, the 50°C isotherm was defined as the lesion boundary. The “ablation” was also terminated when the maximum temperature in the tissue (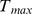) was greater than 100°C.

The main metrics used to evaluate lesion formation at each catheter geometry configuration were lesion volume, depth, and 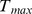.

### Biological Impedance Measurements

A simple tissue model was set up to match the computer model. It comprised the same components: a dispersive patch electrode, a “blood” bath (saline), a tissue slab and an ablation catheter. While this is simple compared to actual left atrial geometry, it allowed us to validate our result quantitatively. However, a frequency of 20 kHz was used since at this frequency, the difference between permittivity and conductivity between tissue and blood was greatest.

The dispersive patch electrode was a stainless steel disc that had a diameter of 16.5 cm and was 6 mm thick. The patch electrode served as the reference, return current path. This was submerged in a saline bath that was 0.9% saline diluted with deionized water until a conductance of 6.5 mS was reached. The tissue was taken from preparations of cow ventricle obtained from the butcher and was not perfused. The tissue slab was placed in the centre of the patch electrode, endocardial side up, and secured with clamps. A 7 French 4 mm irrigated catheter was placed in a mill arm to precisely control its angle and depth. Angles of 0°, 30°, 60° and 90° and depths of 0 mm (in the bath), 2 mm and 4 mm were considered. The depth was measured as the length that the mill arm travelled relative to the point when it just touched the surface.

Each measurement was an average over a three second epoch taken by a USB-6259 data acquisition board and LabVIEW software (National Instruments,TX USA) with a sampling frequency of 2048 Hz. This was done in order to assess the variability of each measurement, giving a sense of consistency and repeatability of each measurement. Thirty-six measurements were taken.

## Results

### Computer Simulations

**Figure 3:**
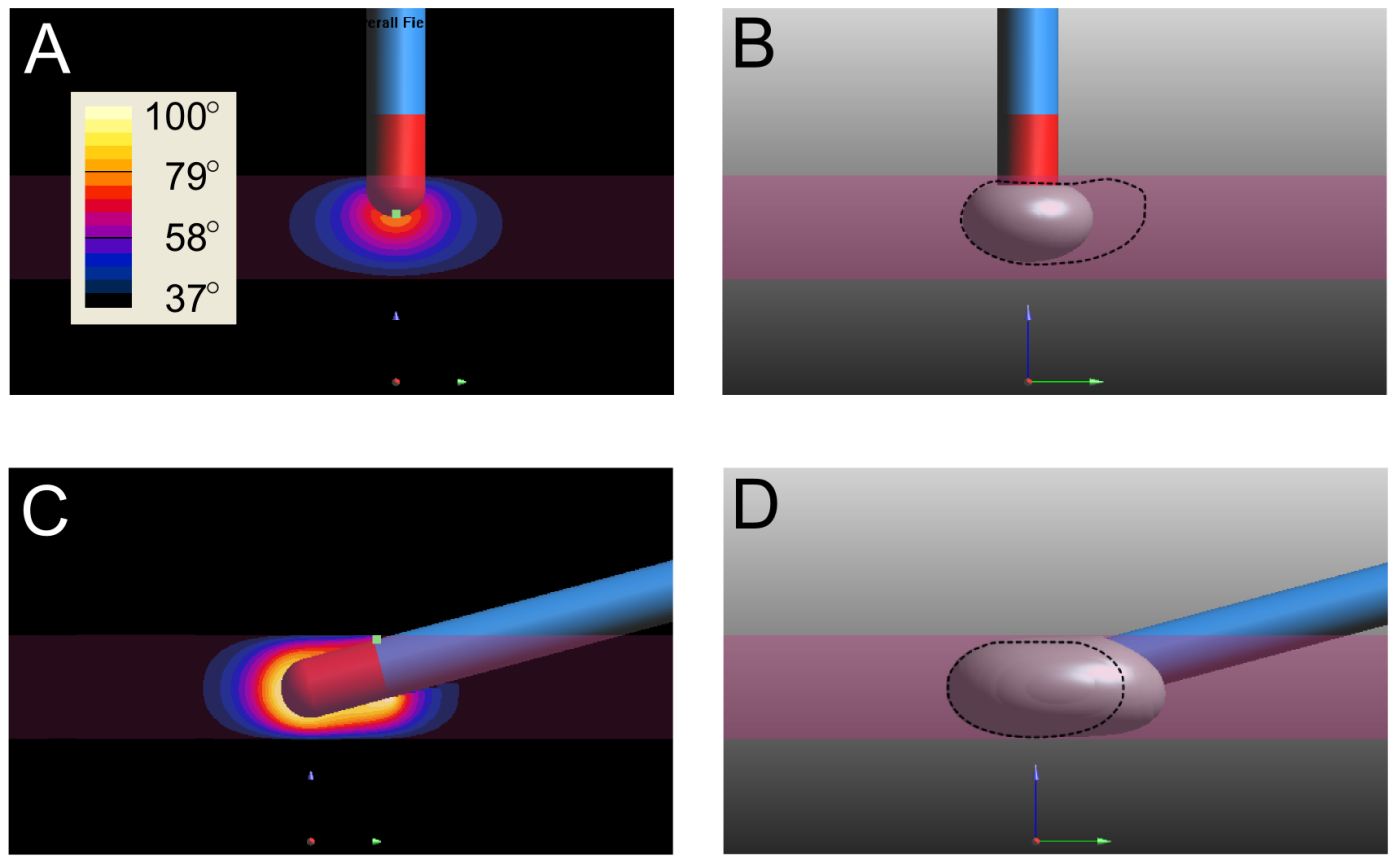
Temperature Distribution Map and Resulting Lesion Volume. For a catheter at 1.6°, temperature (A) and lesion (B) are shown after ablating for 60 s. Dashed volume depicts resulting volume from catheter at 1.6 mm depth at 15° after ablating for 60 s. Temperature distribution map (C) and lesion volume (D) with catheter at 3.2 mm penetration depth and 15° after ablating for 19 s. Dashed volume depicts resulting volume from catheter at 3.2 mm depth and 90° after ablating for 60 s. Legends in A and C show temperature in degrees Celsius.

Four different catheter configurations are presented in Figure 3, each resulting in different end lesion sizes (data for all studied configurations can be found in Table 3). Figure 3A shows the temperature distribution map when the catheter is 1.6 mm deep, at 90° and after 60 s of ablation. We see that there is a small region of higher temperatures ∼75 °C below the catheter tip and the temperatures decay radially. The resulting lesion volume of this temperature distribution is shown in Figure 3B. The dashed line shows the outline of the lesion resulting from a 15° ablation at the same depth. Interestingly, the lesion is deeper (3.480 mm vs. 2.924 mm), wider (6.971 mm vs. 5.223 mm) and has greater volume (72.71 mm^3^ vs. 43.22 mm^3^) at 15° than at 90° despite using less power (5.61 W vs. 6.15 W).

**Table 3:** Lesion parameters at simulation end for all depths and angles for computer simulations.

Depth	Angle	Ave. Power	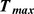	Lesion Width	Lesion Depth	Lesion Volume
mm	°	W	°C	mm	mm	mm^3^
−4	All	7.68–7.74	37.70–37.90	0	0	0
0.04	All	6.86–7.68	46.44–47.12	0	0	0
0.8	90	6.62	59.38	3.165	1.772	10.15
	75	6.61	58.76	2.958	1.768	9.272
	60	6.55	59.22	3.080	1.816	10.26
	45	6.50	58.90	3.182	1.823	10.52
	30	6.40	58.88	3.374	2.137	11.83
	15	6.29	58.52	4.209	2.097	15.56
1.6	90	6.15	77.16	5.223	2.924	43.22
	75	6.10	77.16	5.135	2.912	43.63
	60	6.04	77.16	5.294	2.909	44.69
	45	5.93	77.11	5.449	2.952	47.47
	30	5.79	77.00	6.026	2.938	52.54
	15	5.61	78.50	6.971	3.480	72.71
2.4	90	5.71	92.81	5.910	3.369	76.51
	75	5.64	93.02	6.275	3.509	77.67
	60	5.53	93.84	6.413	3.561	81.66
	45	5.30	95.21	6.700	3.702	90.93
	30	5.09	97.46	8.125	3.621	109.5
	15	4.96	100	8.174	3.649	121.6
3.2	90	5.30	96.00	5.135	3.553	98.05
	75	5.21	97.05	5.223	3.794	102.4
	60	4.98	99.69	5.294	4.000	112.1
	45	4.76	100	7.789	4.000	120.5
	30	4.58	100	8.125	4.000	120.5
	15	4.35	100	7.805	4.000	116.4
4	90	5.07	86.71	6.734	4	111.4
	75	4.92	91.62	7.361	4.00	117.2
	60	4.81	100	7.456	4.00	115.3
	45	4.70	100	7.389	3.995	108.7
	30	4.63	100	7.713	3.965	112.3
	15	4.70	100	7.270	4.00	117.4

Figure 3C shows the temperature distribution map when the catheter was advanced further into the tissue, 3.2 mm deep, at 15° but after only 19 s of ablation. Once again, there is a region of higher temperatures (almost reaching 100°C) surrounding the catheter tip and following the central axis of the catheter. The resulting lesion volume is shown in Figure 3D. This time, the dashed line shows the outline of the resulting lesion from a 90° ablation at the same depth, but, since at 90° the temperatures never quite reach the endpoint (100°C) this is the lesion after a full 60 seconds. Comparing the two configurations, the lesion is deeper (4 mm vs. 3.553 mm), wider (7.805 mm vs. 5.135 mm) and 18.7% more voluminous (116.4 mm^3^ vs. 98.05 mm^3^) at 15° than at 90° despite using less power (4.35 W vs. 5.30 W) once again. Both shallow and deep penetration depths show that despite using less power, there are significant angle related differences in end lesion size and resulting temperatures in the tissue. This is important since many ablations use constant power delivery where this effect would most likely be exacerbated.

**Figure 4:**
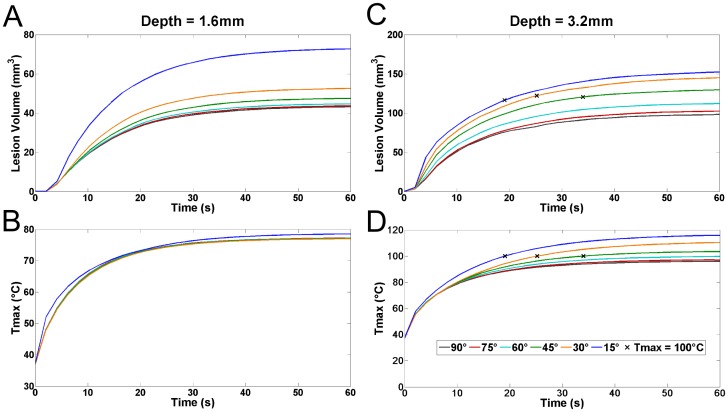
Effects of catheter angle and penetration depth on lesion formation and 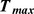. Lesion volume (A) and maximum temperature attained (B) for a penetration depth of 1.6 mm. Lesion volume (C) and maximum temperature attained (D) for a penetration depth of 3.2 mm. Small black ‘x’ marks indicate the time at which 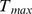 inside the tissue has reached 100°C. Angles in all graphs are given by the legend in (D).

It is also important to study the time course of lesion formation in order to gain insight into temperature development during ablation. In Figure 4 representative plots of the 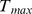 in the tissue and the lesion volume vs. time for two penetration depth cases are shown. Penetration depths of 1.6 mm and 3.2 mm were selected because at depths less than 0.8 mm, heat could not accumulate sufficiently quickly to form lesions, and because they give an idea of just how the 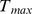 and lesion volume vary according to catheter angle.

Despite having only a small difference in angle and associated 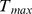 values (≈1.34°C difference) when the penetration depth was 1.6 mm, the final lesion volume was quite a bit bigger for more acute catheter angles; particularly for an angle of 15° where the resulting lesion volume was 72.71 mm^3^ (compared to 43.22 mm^3^ at 90°). Also, it is clear that that steady-state has been reached spatially meaning that at the boundaries of the lesion, thermal equilibrium has been reached and that further application of current will not grow the lesion any more.

At increasing penetration depth (at 3.2 mm penetration depth, compare Figures 4A,B to Figures 4C,D), the angle dependant temperature spread increased and so did the resulting lesion volume. At this increased penetration depth we also saw temperatures exceed the chosen study endpoint of 100° at more acute catheter angles, the first of which (at 15°) happens after only 19 s, but at more obtuse angles the critical temperature was not reached. Of note here is that despite the increased temperatures, if safety implications are ignored with such a rapidly spiking 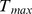, all catheter angles delivered roughly the same lesion size (if the study is terminated when 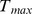 = 100°C).

**Figure 5:**
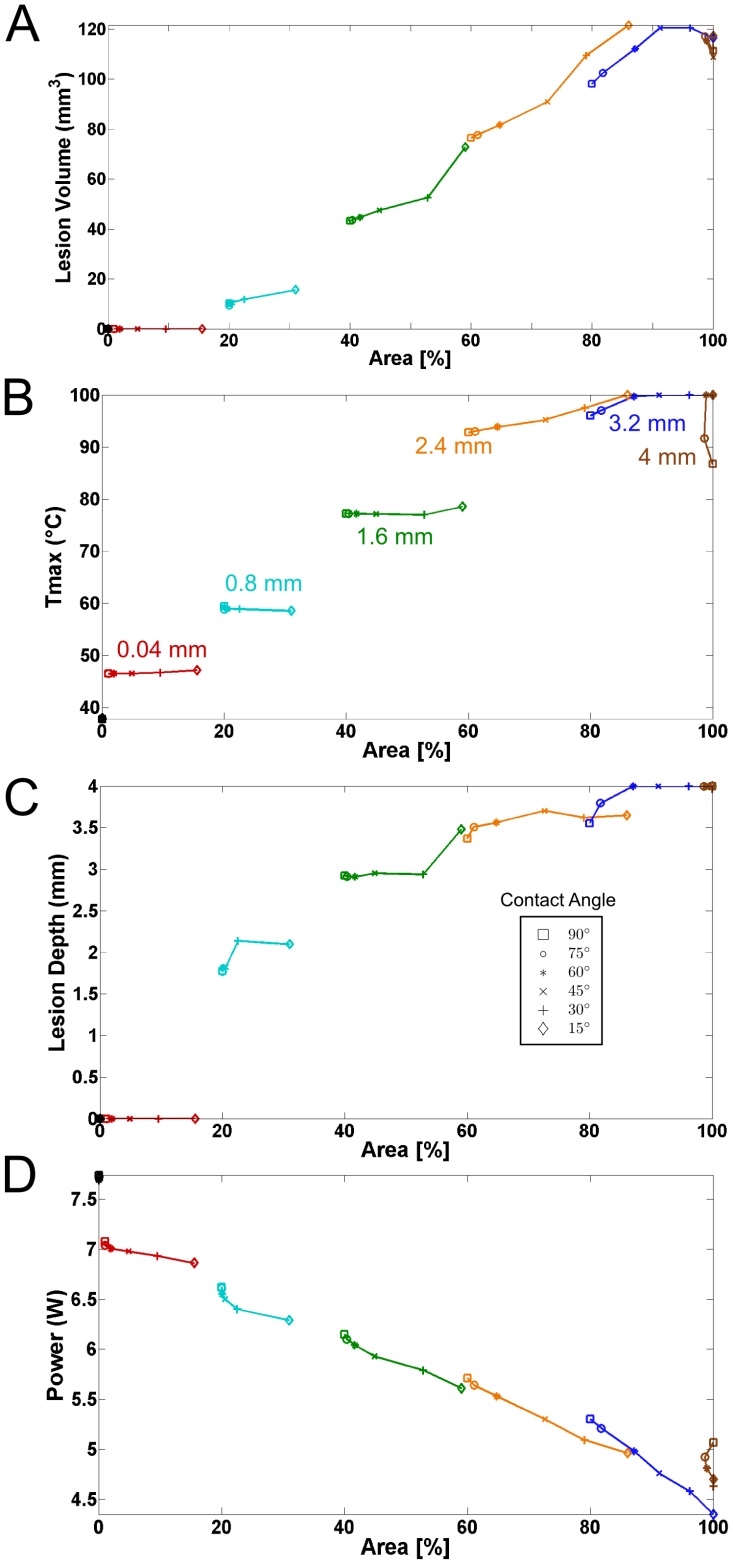
Lesion formation indicators and their relationship with catheter contact area under high flow conditions. Plots show (A) Lesion Volume, (B) 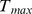, (C) lesion depth and (D) ablation power with respect to catheter contact area. Penetration depth is indicated by color while contact angle is indicated by symbols.

The most important parameters of the lesion were plotted against catheter contact surface area in order to determine whether the angle dependence could be explained by the increase in contact at the different angles. In Figure 5 we can see that the resulting lesion volume and depth increase nearly linearly with contact area. That trend holds with lesion volume until it is artificially truncated by the 100 °C endpoint or the catheter has fully penetrated the tissue at 4 mm.

It can also be observed in Figure 5B that 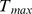 is more dependant on the penetration depth than contact area, with large increases in 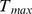 coming every time the catheter is advanced. A final insight is revealed in Figure 5D: with a constant voltage ablation scheme, the true effect that contact has on lesion progression can be observed without compensatory inference which would only have served to complicate the study. In a constant voltage scheme, the average power drawn during an ablation is dependent on the interaction of the catheter with the tissue since increased interaction causes increased impedance. Once again, the exception exists for the case when the catheter is fully penetrating the tissue (4 mm).

### Biologically Measured Impedance

Measurements collected in the 36 impedance trials described above are compiled in Table 4. This data is consolidated to the twelve separate geometries that the 36 trials represent. The recorded impedance when the catheter is in the bath (not engaged with the tissue) is approximately 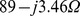 while the model calculates one of approximately 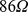; this starting point offers quite good agreement. As the catheter becomes more engaged in the tissue, the impedance increased in magnitude both in its real component (resistance) and imaginary component (reactance becomes more negative), as predicted by the model.

**Table 4:** Consolidated Biological Results.

Depth (mm)	Angle (°)	Resistance ( 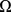 )		Reactance ( 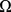 )	
		Mean	Std.	Mean	Std.
0	0	89.52	1.242	−3.464	0.0828
	30	88.55	5.146	−3.401	0.5104
	60	88.79	2.724	−3.312	0.3711
	90	87.47	2.451	−3.249	0.2213
2	0	112.2	4.855	−6.018	0.6260
	30	106.0	6.252	−5.395	0.8053
	60	103.8	1.712	−4.972	0.1090
	90	107.2	1.757	−5.542	0.3165
4	0	121.3	5.879	−7.192	0.6623
	30	117.3	1.626	−6.842	0.3022
	60	123.8	1.667	−7.884	0.4591
	90	121.4	10.69	−7.664	1.615

During each measurement, 6000 samples were taken over three seconds. The standard deviation for each epoch was relatively low (less than 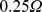 resistance and 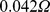 reactance). Thus, for each sample, the measured impedance did not fluctuate very much and was repeatable. In Table 4 we can see that the standard deviation of measurements taken between tissue preparations was an order of magnitude higher. This was probably due to the extremely low sample size, only three per category, since the variability did not seem to be related to the depth or the angle.

**Figure 6:**
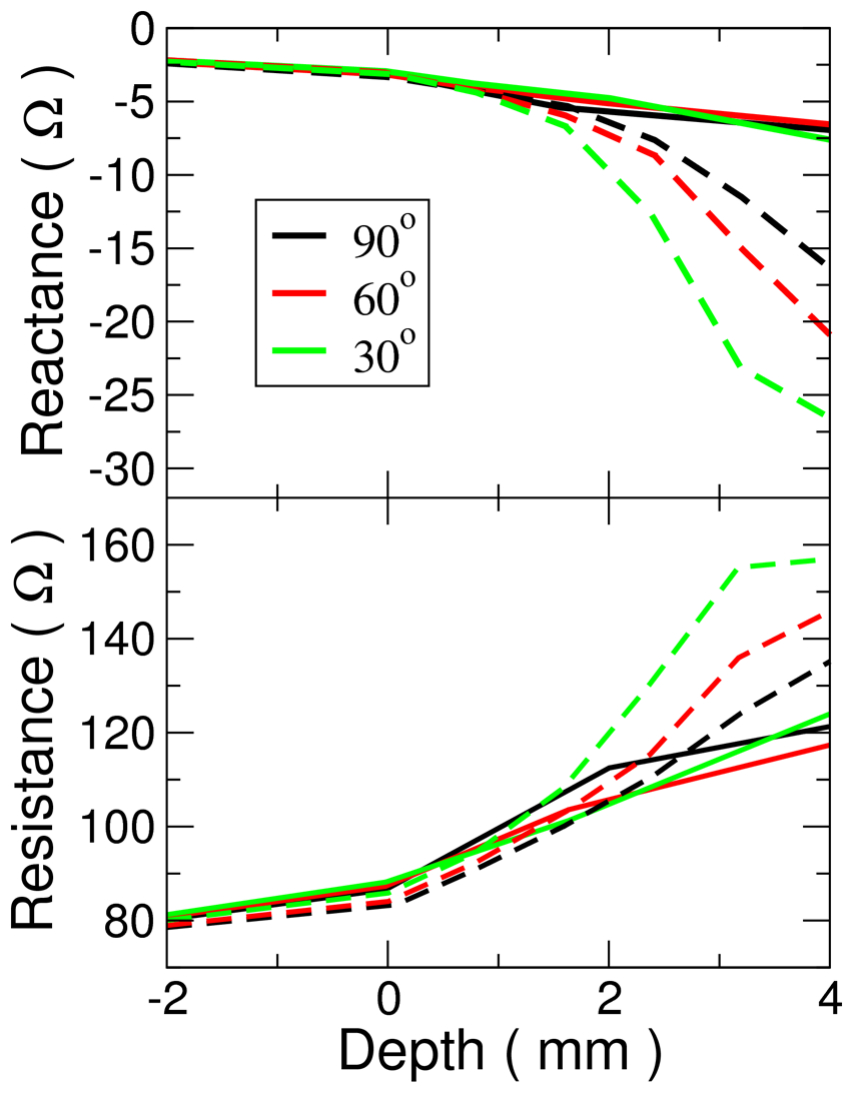
Comparison of biological and simulated impedance. (Top) Electrical reactance as a function of insertion depth. (Bottom) Electrical resistance as a function of insertion depth. Simulated values are given by dashed lines while solid lines indicate biological results. Color indicates the same angle for both simulations and biological experiments.

Biological impedance measurements were compared with simulated ones (see Fig. 6) The simulated impedances matched the experimentally measured ones well for penetration depths up to 2 mm with resistance matching better than reactance, and higher angles matching better than lower ones. Outside of the tissue, the impedance differed by less than one percent between simulation and experiment. At 2 mm, the maximum resistance error was 10% while it was 100% for reactance. At 4 mm, the maximum errors for resistance and reactance were much worse, being 27% and 353%, respectively. Simulation trends agreed with biological experiments: reactance became more negative and resistance increased with insertion depth; however, a great dependence on angle was not observed.

## Discussion

This study presents a detailed model of catheter heating of myocardial tissue. It is the first to consider catheter contact angle in the context of ablation, and with time dependent lesion formation. The time course is highly significant because the maximum temperature of 100°C may be reached before steady state is reached. At this temperature, serious complications occur and ablation must stop. Thus, previous studies ignoring the evolution of the lesion may have overestimated lesion volume.

Catheter angle had a very large effect on lesion volume with up to a two-fold difference in size between the angle extremes. As the angle between the catheter and the tissue became more acute for a given depth, more electrode surface was in contact with myocardium. Thus, this easily explains a larger lesion volume without increasing lesion depth. However, lesion depth also increased greatly for very acute angles, due to a larger heating surface having less curvature directly under the electrode.

It is clear that catheter contact geometry (not just penetration depth) is important not only when it comes to the ultimate lesion dimensions but also to the 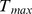 induced in the tissue. At greater penetration depths, catheter angle played an even more significant role in lesion formation parameters with the more acute angles (particularly 15°) having the greatest depths, widths and volumes. While it was found that a minimum penetration depth of 0.8 mm was required for thermal injury to occur, transmural or close to transmural lesions were not recorded until the penetration depth was between 2.4–3.2 mm.

Ablations were considered at the same penetrations depths but the angles were varied and the resulting lesion volumes, lesion depths and average electrical powers were often significantly different. Interestingly, 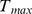 varied more according to the depth of the catheter than to the specific contact area. The magnitude of all of these findings is compounded by the fact that the ablation power was inversely proportional to contact area. This is particularly significant given that one of the primary ablation schemes is constant power. Obviously, when using a constant power scheme, increases in catheter contact area (and the corresponding increase in impedance) have no impact on the output power; unlike when using a constant voltage ablation scheme where an increase in impedance leads to decreased output power. By holding power constant, the power delivered at higher contact levels would be greater than that shown in this study (which are inversely proportional to impedance). Another oft-proposed scheme, constant current ablation would further intensify the problem since the power delivered at higher contact levels would be greater still (since it is now directly proportional). From these differences, it is reasonable to infer that there would be larger lesion volumes (consistent with in-vitro studies), depth and 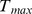 in constant power and current schemes compared with constant voltage due solely to increasing catheter contact area, which the operator would probably be unaware of.

It is important to consider that small changes in contact would be virtually undetectable by standard contact assessment techniques which could result in large changes in not only lesion parameters, but also in the heating rate. Standard clinical ablation catheters typically have an embedded temperature sensor measuring temperature of the tip; however, 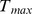 was never located at the catheter/tissue interface; in fact, the tip surface temperature differed from 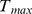 by as much as 10°C. This finding is easily explained and strongly dependent on the flow rate on the endocardial surface: the convection rate on the endocardial surface dissipates heat much quicker than it can be conducted away within the tissue; leading to observed 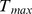 values spatially located below the catheter and well into the tissue. This finding is well known in ablation research and lead Kongsgaard et al. to conclude “...the tissue temperature may far exceed the catheter tip temperature, and intramyocardial superheating resulting in steam formation and popping may occur despite a relatively low catheter tip temperature.” [26]. This discrepancy between catheter tip temperature and 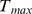 can be particularly exacerbated when irrigated catheters are used. These catheters manage the surface temperature well but it was observed that “Catheter tip and tissue temperatures were markedly discrepant” [27], once again making it difficult for the operator to know for sure whether adequate contact/lesion formation or tissue overheating is occurring. Our finding of 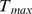 is also supported by a study of lesions in canine muscle where higher temperatures were found several millimeters into the tissue[28]. Often, before equilibrium is reached, the tissue heats up very rapidly and any compensatory mechanisms may not be able to react appropriately since they are measuring surface temperatures. It is clear that more work must be done to understand this key procedural input particularly as current ablation techniques involve using either constant power or temperature controlled ablation.

No special consideration was given to the mechanical deformation of the tissue that occurs as a result of engagement with the catheter. Using a homogeneous slab of tissue, such as the one modelled in this study, there would be a certain amount of mechanical compliance observed, meaning that when the catheter was pressed into the tissue, the tissue would deform and with a sloping pit around the tip of the catheter, particularly at deeper insertion depths. Not deforming the tissue leads to artificially high contact percentages. With a real tissue slab, it would be very difficult to imagine a scenario where the catheter tip was fully engaged with the endocardial tissue other than if it somehow punctured it. However, the left atrial endocardium is quite complex. Different tissue thicknesses, fiber orientations and trabeculated structures abound. Therefore, the modelled scenario, increasing catheter contact without mechanical deformation of the tissue, is quite plausible as the catheter tip could go into a natural groove of the left atrial wall. Ironically, it is the high degree of tissue inhomogeneity that makes the choice to implement a homogeneous slab of tissue and disregard its mechanical compliance such an apt one.

Catheter contact has been shown to have a significant impact on lesion formation. While other works in the field acknowledge that this may be the case, this is the first investigation into catheter angle contribution. Given a demonstrated difference in lesion sizes attributable to changes in contact, this is a particularly interesting avenue to explore further, particularly when its potential impact on more sophisticated modern ablation techniques is examined. Irrigated catheters have long been in use as a means to cool the surface and allow heat to propagate more deeply into the tissue, resulting in larger lesions. It is therefore interesting to consider the interactions that would occur in scenarios considered in this paper where a more acute catheter angle leads to more rapid heat accumulation. Irrigation allows more time to pass before the tissue temperatures reach dangerous levels but doesn't change the electrical coupling of the catheter to the tissue. This study has shown a strong correlation between ablation power and catheter contact and an improved ablation modality may factor in this new information.

### Comparison with biological tissue

Simulation results were not in good agreement quantitatively with biological experiments for tissue depths greater than 2 mm. The primary effect responsible for this is probably the mechanical deformation of the tissue. Tissue was modeled as being liquid, that is, fully enclosing the electrode up to the insertion depth. Experimentally, a more gradual sloping pit probably formed around the electrode tip reducing contact area. Less contact with tissue allows more current to flow into the bath, and reduces resistance.

Our simulations show an exponential dependence of contact area with impedance. It is likely that the ventricular tissue studied is stiffer than atrial tissue in situ for several reasons: 1) The ventricular tissue is thicker than atria tissue with a more complex laminar organization to withstand higher pressures; 2) the tissue was backed by a rigid metal plate whereas atrial tissue can deform until constrained by the pericardium; and 3) post-mortem changes. This hypothesis is consistent with the theoretical ones which also show that when only the hemispherical tip is in contact with the tissue, little angle dependence of impedance variations are observed.

Additionally, the experimentally measured reactance did not agree as well with the model as the resistance. Differences in material properties accounted for some of this deviation. The conductance of the saline bath was adjusted to that of blood by diluting with saline but, the permittivity of water is quite different from that of blood[29], leading to a different reactance.

Despite these differences at deeper penetration depths, the agreement at shallow depths demonstrated that the simulations results were reasonable and within plausible physiological range. For simulations at 485 kHz, our results match well with previously published values[30]. It also underscores the dependency of lesion formation on the contact geometry. Our simulations can be taken as the extreme end of the spectrum, a “liquid tissue”, while the biological experiment is the other end of the spectrum, constant hemispherical contact. Given the lack of rigid support around the atria and an endocardial surface with a high degree of small trabeculations, we feel the actual situation is closer to the simulation results presented.

### Model limitations

Although we have tried to include many factors in these simulations, there are still several simplifying assumptions that have been made. Foremost, we have assumed the complete catheter tip up to the insertion depth is in contact with the tissue. Actual biological tissue is stiffer and as we saw with the biological experiments, this would lessen the angle dependence of lesion formation. Another effect of stiffer tissue would be to allow more fluid flow near the electrode, leading to a cooler tip, moving 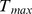 further into the tissue, and increasing the discrepancy between 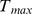 and the electrode surface temperature. However, given the highly deformable and trabeculated nature of the tissue, our approach is justified. The nature of the contact is not controllable and so may vary between the two extremes presented.

We have ignored tissue anisotropy, which is not well characterized in the atria. Electrical current prefers to flow along the axis of myocytes. In the left atrium, tissue is composed of a limited number of layers with myocyte direction changing markedly between them[31]. Experiments have measured a low degree of anisotropy[32] which may be due to homogenizing effect of the tissue structure, or gap junctions which are distributed more equitably between longitudinal and transverse directions.

## Conclusions

This study makes several important discoveries regarding the role that catheter contact angle/penetration depth combinations play in lesion formation and maximum temperature induced in the tissue. It was found that more acute catheter angles caused larger lesions than their more obtuse counterparts (even at the same penetration depth) and this effect was magnified as the catheter pushed deeper into the tissue. For the most part, the end lesion dimensions could be predicted based on catheter surface contact area.

Of clinical importance is the fact that the catheter contact is unknown and largely uncontrollable due to the complex anatomy found in the left atrium and the imprecise nature of situating the catheter. Given the impact of catheter contact that we have demonstrated on all aspects governing success of the procedure, it is clear that modifications should be made to any currently used ablation scheme (i.e.- constant power, constant temperature) to include a some electrical indicator of catheter contact whether it be impedance or power.
